# Rupture of Diaphragm

**Published:** 1901-06

**Authors:** 


### RUPTURE OF DIAPHRAGM.

Dr. J A. Thornborrow, of Virginia, Ill., reports the death of a mule from rupture of the diaphragm and strangulated hernia of the thorax. The rupture was on the left side about the middle of the diaphragm and midway of the superior-inferior diameter, and about the size of a silver dollar, through which at least some ten feet of the intestines had passed into the thoracic cavity. The animal had been in its usual condition the day previous, and until within six hours of its death had shown no unusual signs.

